# Frequent use of online medical records: analysis of influence factors based on structural equation modeling

**DOI:** 10.3389/fpubh.2025.1609503

**Published:** 2025-08-20

**Authors:** Wei Wang, Lei Qin, Yang Chen, Yinzhi Wang, Linglong Ye, Ruojia Wang, Yingqiu Zhu

**Affiliations:** ^1^School of Economics and Management, Guizhou Normal University, Guiyang, China; ^2^School of Statistics, University of International Business and Economics, Beijing, China; ^3^Dong Fureng Institute of Economic and Social Development, Wuhan University, Wuhan, China; ^4^Petrochina (Beijing) Digital Intelligence Research Institute CO., LTD, Beijing, China; ^5^School of Finance, Shanghai University of International Business and Economics, Shanghai, China; ^6^School of Public Affairs, Xiamen University, Xiamen, China; ^7^School of Management, Beijing University of Traditional Chinese Medicine, Beijing, China

**Keywords:** online medical records, influence factors, structural equation modeling, HINTS, public health

## Abstract

The advent of electronic storage of medical records and the internet has led to an increase in the use of online medical records, thereby enhancing doctor–patient communication and facilitating medical treatment. Based on demographic and personal behavioral characteristics from the National Cancer Institute’s 2019–2020 National Trends in Health Information Survey data, this study explored the characteristics and factors influencing the frequent use of online medical records and compared them with those that do not. By combining traditional statistical tests and two machine learning algorithms, eight variables were identified as key variables in the frequent use of online medical records. These variables were then divided into three influencing factors (latent variables). The structural equation model was used to conduct impact path analysis of the three influencing factors and target variables. The three impact factors were (1) Whether to provide online medical records, (2) Degree of concern for health, and (3) Whether to use internet. This paper proposes recommendations based on the three impact factors, thereby promoting the usefulness of medical records in a larger group of people.

## 1 Introduction

As a key tool, online medical records (OMR) help individuals and caregivers in understanding their health and managing their healthcare needs (1). Guo et al. (2) pointed out that online medical systems can improve the operating efficiency of hospitals. Derecho et al. (3) discussed the contributions to the development of online medical records from the perspective of physicians. Fleming et al. (4) highlighted that online medical records can be integrated with large language models to enhance the efficiency of hospitals. A regular review of self-medical records by patients can enhance doctor–patient communication. Such an effective communication can increase patient satisfaction and acceptance of medical services. More importantly, it can enhance patient compliance and cooperation with medical teams (5). Therefore, ensuring good communication by recording, processing, and sharing health information with patients is a must and an integral part of the healthcare process. Lloyd et al. (6) emphasized that promoting the widespread adoption of electronic medical records is fundamental to the digital health system, enabling hospital clinicians to deliver safer and more effective healthcare. The advent of electronic storage of medical records and the internet has allowed patients to access their medical records online. Internet-accessible medical records are more advantageous in terms of readability, accessibility, and popularity than traditional paper medical records written in technical language and containing raw data. Some studies have shown that online electronic medical records can replace traditional medical records (7–9).

While access to OMR is crucial for public health and OMR systems are maturing, many individuals still do not use OMR. The key questions related to this topic that remain unanswered are as follows: (1) What are the characteristics of frequent users of OMR, (2) What factors lead patients to use OMR frequently, and how do these factors affect patient’s selection?

Some studies have investigated the differences in the characteristics of the two groups of people who use OMR and who do not and the influencing factors of the suitability of OMR. However, some deficiencies still exist. First, most existing studies on the non-use of network medical records only include a specific factor and a specific population and are not sufficiently comprehensive. For example, a study investigated the relationship between social media use and whether OMR are used (10), and another study investigated the association between whether access is provided and whether OMR are used (11). Second, many studies have only used traditional statistical algorithms to discuss the correlation between influencing factors and target variables. Moreover, they did not discuss the relationship between the factors nor did they discuss the path of influence. Therefore, no clear policy recommendations can be implemented to increase OMR use. Based on the Health Information National Trends Survey data, Elkefi et al. (12) analyzed the characteristics and differences between individuals who use OMR and those who do not. This study found significant differences in social factors such as gender, race/ethnicity, and age between the two groups. The study considered more factors and used more authoritative databases. However, only the correlation between each influencing factor and the target variable was discussed, whereas the influence path was not explored. Finally, the current study also divided the population into two groups: those who use OMR and those who do not. However, people who have used OMR may not be “frequent users,” and therefore, the “non-user” and “frequent user” of this difference between the two groups must also be studied. The current study also did not perform this subgrouping.

Considering the limitations of existing research on OMR usage, this study made some corresponding improvements. First, data for this study were drawn from the National Cancer Institute’s 2019–2020 Health Information National Trends Survey (HINTS). HINTS is an ideal authoritative data source for providing an in-depth examination of the non-user characteristics of OMR (10). Furthermore, it can provide a variable-rich environment for finding influencing factors. Second, this study first used the traditional hypothesis testing method to explore the correlation between many influencing factors and target variables and performed preliminary screening of variables according to the test results. Then, machine learning models were used to determine the final influencing factors. Later, the structural equation model was used to discuss the influence path between the influencing factors and the target variable, as well as the interaction between the influencing factors. Based on the influence path, policy recommendations for increasing the use of OMR are put forward. Finally, this study divided the population into “frequent user” and “non-user” groups based on the OMR use frequency and explored the characteristics of OMR frequent users compared with non-users.

In analyzing the influencing factors of OMR usage, this work first identifies three core factors that affect the frequency of use through empirical analysis: system accessibility (whether to provide OMR), user health awareness (degree of concern for health), and technology acceptance (whether to use Internet device). This finding addresses a gap in the existing literature, which tends to emphasize technical aspects (such as system functionality) while neglecting individual user differences. It offers a more comprehensive perspective for understanding the driving mechanisms behind electronic medical record usage behavior. Furthermore, unlike traditional research that primarily focuses on factors related to medical institutions, this study innovatively incorporates patients’ subjective psychological characteristics (health concerns) and technology use capabilities (technology acceptance) into the analytical framework. The results indicate that even when a hospital provides a comprehensive electronic medical record system, individual patients’ cognition and attitudes remain crucial in determining usage frequency. This finding provides a theoretical foundation for a “patient-centered” approach to medical information construction.

The remainder of this paper is organized as follows. Section 2 outlines the data sources and variable selection. Section 3 describes the model and analysis methods. Section 4 presents the results of the empirical analysis, while Section 5 offers further discussion on these results. Finally, Section 6 concludes this work.

## 2 Methods

### 2.1 Data source

Study data were collected from the National Cancer Institute’s 2019–2020 HINTS. HINTS is a nationally representative survey of the US non-institutionalized adult population that collects data on the American public’s need for, access to, and use of health-related information (13). Note that the HINTS data did not include only cancer patients. This study analyzed merged data from Cycle 3 to Cycle 4. Data from Cycle 3 were collected between January and May 2019 and those from Cycle 4 were collected from February 2020 to June 2020. The respondents were screened on the basis of the target-dependent variable (i.e., frequent use of OMR), leaving the respondents with no missing values in the target-dependent variable. Finally, 6,380 respondents were screened out (*N* = 6,380). Among them, 971 individuals used OMR frequently and 5,409 individuals who never used OMR. HINTS administration was approved by the Institutional Review Board at Westat Inc. and deemed exempt by the National Institutes of Health Office of Human Subjects Research. HINTS data are available for public use. Additional information on the survey design is available on the HINTS website.

The target variable “Frequent Use of OMR” in this study was calculated on the basis of the following question in HINTS: “How many times did you access your online medical records in the last 12 months?” Questionnaire participants have options to choose from “0,” “1–2 times,” “3–5 times,” “6–9 times,” and “≥10 times,” and its corresponding variable name was “Times of Access to OMR.” We used this question to identify “frequent users” and “non-users” of OMR. The respondents who reported accessing their records “6–9 times” or “>10 times” were coded as “frequent users,” and those who reported accessing their records 0 times were coded as “non-users.”

Sociodemographic variables of interest (dichotomized for analyses) included gender (male, female), race/ethnicity (non-Hispanic white, racial, and ethnic minority), education (≤high school, >high school), income ranges (<$20,000, ≥$20,000), residential area (non-metropolitan, metropolitan), and marital status (with spouse, without spouse), as well as numerical sociodemographic variables, including age (continuous years) and body mass index (BMI).

For further analysis, we selected as many variables as possible from the HINTS database to identify their relationship with OMR use. Almost all variables in the questionnaire were analyzed using statistical tests, including chi-square tests for categorical variables and two-tailed *t*-tests for continuous variables. The 44 variables that passed the significance test were divided into the following blocks according to the questionnaire content (as shown in Table 1): part A (looking for health information), part B (using the internet to find information), part C (your health care); part D (medical records), part E (caregiving), part F (your overall health), part G (health and nutrition), part H (physical activity and exercise), part K (tobacco products, this part of the questionnaire was about the respondents’ diet of tobacco products), part L (cancer screening and awareness), part M (your cancer history), and part N (beliefs about cancer), and a numerical variable.

**Table 1:** Distribution of the characteristics of the variables in the HINTS database.

Variable	Online medical records non-users *n* (%)	Online medical records frequent users *n* (%)	*p-*Value
Categorical variables			
Total	5,409 (84.78)	971 (15.22)	
Socio-demographic variables			
Gender			
Male	2,196 (45.23)	323 (35.22)	<0.0001
Female	2,659 (54.77)	594 (64.78)	
Race			
Non-Hispanic white	3,492 (71.48)	753 (81.58)	<0.0001
Racial and ethnic minority	1,393 (28.52)	170 (18.42)	
Education			
>High school	3,471 (66.60)	841 (88.43)	<0.0001
≤High school	1,741 (33.40)	110 (11.57)	
Income			
≥$20,000	3,569 (75.28)	89 (10.03)	<0.0001
<$20,000	1,172 (24.72)	798 (89.97)	
Income feelings			
Not difficult	3,803 (75.61)	756 (81.03)	0.0003
Difficult	1,227 (24.39)	177 (18.97)	
Area			
Metropolitan	4,728 (87.41)	872 (89.80)	<0.0001
Non-metropolitan	681 (12.59)	99 (10.20)	
Marital status			
With spouse	2,481 (47.73)	617 (64.88)	<0.0001
Without spouse	2,717 (52.27)	334 (35.12)	
A: looking for health information			
Confident_get health info			
High_level	4,796 (90.78)	925 (95.66)	<0.0001
Low_level	487 (9.22)	42 (4.34)	
Trust_doctor			
High_level	4,932 (93.53)	940 (97.01)	<0.0001
Low_level	341 (6.47)	29 (2.99)	
Trust_gov			
High_level	3,372 (68.33)	739 (76.98)	<0.0001
Low_level	1,563 (31.67)	221 (23.02)	
Trust_religious orgs			
High_level	1,501 (30.42)	197 (20.61)	<0.0001
Low_level	3,433 (69.58)	759 (79.39)	
Seek cancer info			
Yes	2,378 (44.50)	682 (70.45)	<0.0001
No	2,966 (55.50)	286 (29.55)	
B: using the internet to find information			
Internet_use			
Yes	3,841 (71.41)	940 (97.21)	<0.0001
No	1,538 (28.59)	27 (2.79)	
Electronic_use			
Yes	3,752 (70.18)	959 (99.38)	<0.0001
No	1,594 (29.82)	6 (0.62)	
Have device			
Tablet computer	342 (6.50)	25 (2.60)	<0.0001
Smartphone	1,579 (30.01)	246 (25.55)	
Basic cell phone only	775 (14.73)	30 (3.12)	
None	384 (7.30)	4 (0.42)	
Multiple devices selected	2,182 (41.47)	658 (68.33)	
Wearable device_track health			
Yes	976 (18.27)	349 (6.24)	<0.0001
No	4,367 (81.73)	614 (63.76)	
Shared health info_device			
Yes	593 (11.20)	346 (36.04)	<0.0001
No	4,076 (76.98)	554 (57.71)	
Not applicable	626 (11.82)	60 (6.25)	
Social media use			
Yes	3,337 (63.09)	858 (88.82)	<0.0001
No	1,952 (36.91)	108 (11.18)	
C: your healthcare			
Regular provider			
Yes	3,285 (62.06)	847 (88.23)	<0.0001
No	2,008 (37.94)	113 (11.77)	
Freq go provider			
None	990 (18.51)	24 (2.48)	<0.0001
1 time	788 (14.73)	32 (3.31)	
2 times	1,031 (19.27)	96 (9.92)	
3 times	791 (14.79)	110 (11.36)	
4 times	686 (12.82)	167 (17.25)	
5–9 times	648 (12.11)	284 (29.34)	
10 or more times	415 (7.76)	255 (26.34)	
Insurance			
Yes	4,288 (92.35)	852 (98.61)	<0.0001
No	355 (7.65)	12 (1.39)	
D: medical records			
Maintained OMR			
Yes	3,829 (71.29)	951 (98.35)	<0.0001
No	220 (4.10)	2 (0.21)	
Do not know	1,322 (24.61)	14 (1.45)	
Access to OMR_provider			
Yes	1,833 (34.37)	935 (96.69)	<0.0001
No	2,429 (45.55)	21 (2.17)	
Do not know	1,071 (20.08)	11 (1.14)	
Access to OMR_insurer			
Yes	715 (15.05)	487 (53.46)	<0.0001
No	2,640 (55.57)	259 (28.43)	
Do not know	1,396 (29.38)	165 (18.11)	
E: caregiving			
Caregiving who			
Yes	723 (13.86)	203 (21.32)	<0.0001
No	4,493 (86.14)	749 (78.68)	
F: your overall health			
Talk health friends			
Yes	4,093 (76.68)	864 (89.91)	<0.0001
No	1,245 (23.32)	97 (10.09)	
Medical history			
Yes	3,414 (64.02)	691 (71.90)	<0.0001
No	1,919 (35.98)	270 (28.10)	
Psychology distress			
Yes	2,657 (51.16)	431 (45.18)	0.0008
No	2,537 (48.84)	523 (54.82)	
G: health and nutrition			
Notice calorie info			
Yes	2,125 (40.07)	550 (57.17)	<0.0001
No	3,178 (59.93)	412 (42.83)	
Drink days per week			
None	2,623 (55.90)	438 (48.08)	<0.0001
1–3	1,389 (29.60)	327 (35.89)	
4–7	680 (14.49)	146 (16.03)	
H: physical activity and exercise			
Moderate exercise			
None	1,661 (31.29)	230 (24.01)	<0.0001
1–3 days per week	1,828 (34.43)	380 (39.67)	
4–7 days per week	1,820 (34.28)	348 (36.33)	
Strength training			
None	3,100 (58.87)	498 (52.20)	0.00048
1–3 day per week	1,523 (28.92)	328 (34.38)	
4–7 days per week	643 (12.21)	128 (13.42)	
K: tobacco products			
Electronic cigarettes			
relative less harmful	2,194 (42.37)	515 (55.44)	<0.0001
relative more harmful	1,000 (19.31)	199 (21.04)	
I do not know	1,984 (38.32)	232 (24.52)	
L: cancer screening and awareness			
Ever tested colon cancer			
Yes	3,161 (60.67)	657 (69.45)	<0.0001
No	2,049 (39.33)	289 (30.55)	
Heard hpv			
Yes	3,112 (58.83)	763 (79.65)	<0.0001
No	2,178 (41.17)	195 (20.35)	
Heard hpv vaccine			
Yes	2,846 (54.66)	728 (76.31)	<0.0001
No	2,361 (45.34)	226 (23.69)	
M: your cancer history			
Ever had cancer			
Yes	789 (14.90)	209 (21.79)	0.0002
No	4,508 (85.10)	750 (78.21)	
N: beliefs about cancer			
Everything cause cancer			
Strongly agree	1,164 (22.73)	174 (18.22)	0.013
Somewhat agree	2,308 (45.06)	483 (50.58)	
Somewhat disagree	1,023 (19.97)	190 (19.90)	
Strongly disagree	627 (12.24)	108 (11.31)	
Prevent cancer_not possible			
Strongly agree	464 (9.01)	38 (3.99)	<0.0001
Somewhat agree	1,287 (25.00)	182 (19.10)	
Somewhat disagree	1,938 (37.65)	414 (43.44)	
Strongly disagree	1,459 (28.34)	319 (33.47)	
Influence cancer_obesity			
A lot	1,540 (30.35)	378 (40.00)	<0.0001
A little	1,634 (32.20)	356 (37.67)	
Not at all	522 (10.29)	74 (7.83)	
Do not know	1,378 (27.16)	137 (14.50)	

Numeric variables	Mean (SD)	Mean (SD)	*p*-value
Age	58.35 (0.24)	55.64 (0.51)	<0.0001
BMI	28.44 (0.09)	29.00 (0.23)	0.0228
Average time sitting	6.58 (0.06)	7.35 (0.12)	<0.0001
Weekly minutes moderate exercise	171.66 (4.94)	148.09 (6.08)	0.0027

Specific variables and their descriptive statistics are presented in Table 1, and Supplementary Table 1 lists details of some of the aforementioned variables, including sociodemographic variables and some variables adjusted for research needs with the readjustment information.

### 2.2 Models

**Figure 1:**
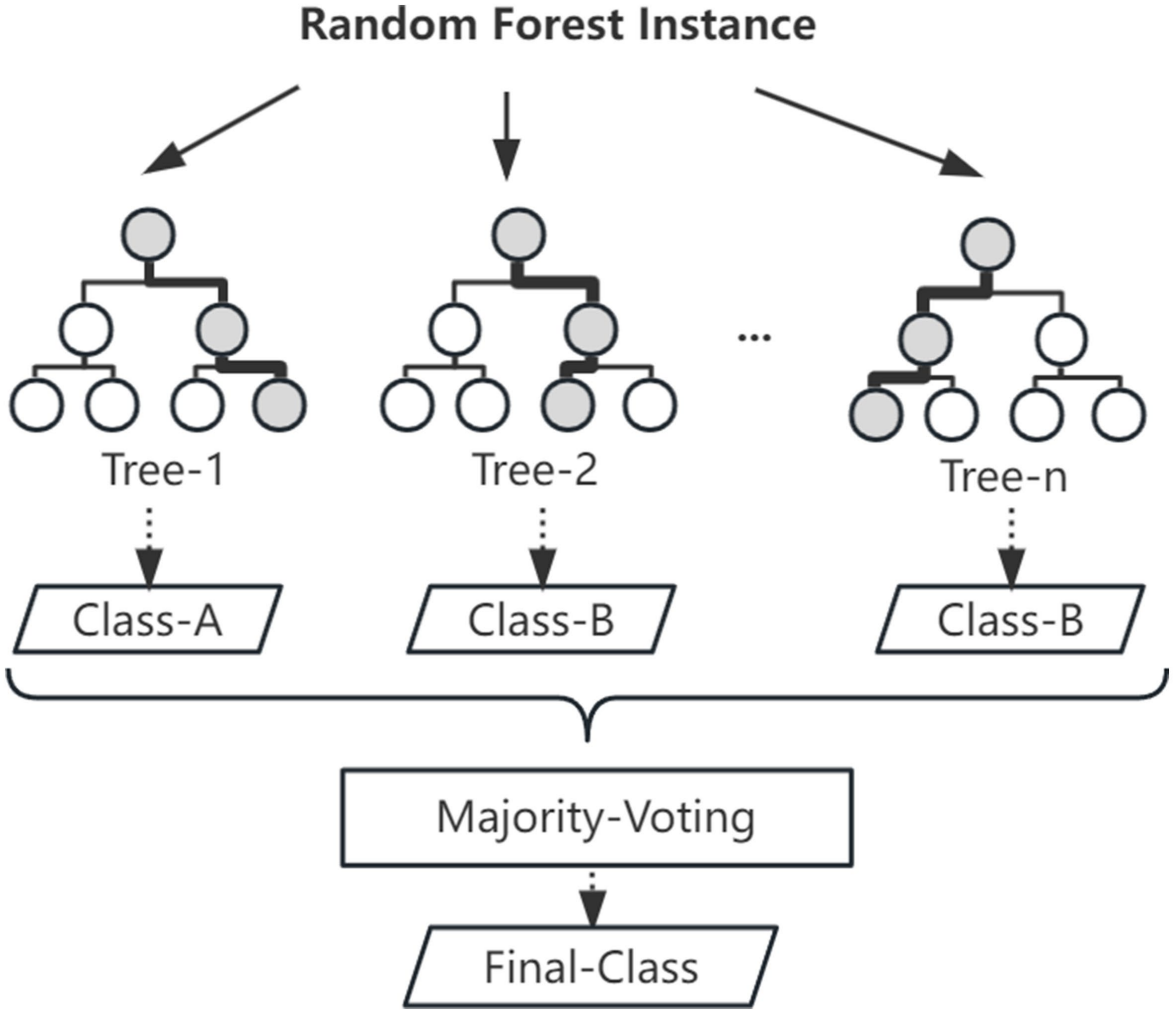
Structure of random forest.

The models used in this work include three types: random forest (RF), generalized linear model (GLM) and structural equation model (SEM). RF, first proposed by Breiman (14), is an algorithm that integrates multiple trees through the idea of ensemble learning. This is a powerful classification and regression tool. The basic unit in a RF is a decision tree that relies on an independently sampled random vector as a weak learner built on a randomly generated training set. In RFs, the final result obtained through the average prediction of all trees for categories or values. The parameters of the final model were as follows: the number of trees was 500, the number of variables contained in the variable selection set at the tree node was 4, and the maximum tree depth was 20 (see Figure 1).

The generalized linear model is a class of regression models that can model outcomes following exponential distributions (15). Compared with the simple linear model, the dependent variable *Y* in the GLM is transformed, and the transformed *Y* has a linear relationship with *X*.


$$f(E(y_{i}))=x_{i}^{T}\beta$$

In the aforementioned formula, *β* are the coefficients of the independent variables *x_i_*, and *f*(•) is the link function. In addition to the Gaussian distribution, the link function can also be established according to the Poisson, binomial, and gamma distributions. Each serves a different purpose, and depending on the type of distribution and link function, can be used for prediction or classification. The GLM model constructed in this study is a regularized GLM that introduces *L*_1_ and *L*_2_ penalty terms to avoid model overfitting and reduces the variance of prediction errors. The link function *f*(•) is the identity, and density corresponds to a normal distribution. Ours is the simplest GLM but has many uses and several advantages over other types of GLMs. The “H2o.grid” function in the “h2o” package was used to conduct grid search on several *α* values set on the (0, 1) interval, and an automatic *λ* search was performed for each *α*. The final selected model had a regularization parameter of 0.05.

Structural equation modeling is a method used for establishing, estimating, and testing causal models. Structural equation models typically include measurement and structural models. In these models, measurement equations describe the relationship between latent variables (variables believed to exist but cannot be directly observed) and observable variables. On the other hand, structural equations describe the relationship between latent variables. A structural equation can also be used to discover the relationship between multiple explanatory variables and the explained variables; the variables involved are typically numerical variables. However, measurement variables mostly collected from the questionnaire are categorized variables, and therefore, conventional MLE is no longer applicable. Instead, weighted least squares estimation with adjusted mean and variance (WLSMV) can be adopted through Mplus.

As suggested by Anderson and Gerbin (16), confirmatory factor analysis models were tested first, followed by a fit of structural models to explore the hypothesized relations among the variables of interest. The WLSMV was used to estimate the fit of a model, and standardized path coefficients (SPC) and their *p* values from the hypothesized model were used to test different study hypotheses. To evaluate model quality, several fit indices were also reported for the overall models, including the Tucker–Lewis Index (TLI), Comparative Fit Index (CFI), and root mean square error of approximation (RMSEA). Traditionally, models with TLI and CFI indices of >0.9 are considered to have a relatively good fit of the data (17), and an RMSEA of >0.10 indicates a poor fit (18). Relative goodness-of-fit indices (e.g., CFI, TLI) measure the fitting results of the structural equation. It represents the improvement in the goodness-of-fit of the hypothesized model relative to the baseline model. To calculate the relative goodness-of-fit indices, statistical values and degrees of freedom of the hypothetical and baseline models are required, namely *T_h_*, *df_h_*, *T_b_*, and *df_b_*. One of the key problems associated with relative goodness-of-fit indices is that the baseline model is absurdly limited, whereas absolute goodness-of-fit indices (e.g., RMSEA) do not depend on the arbitrary baseline model. RMSEA is an indicator of lack of fitting, the larger the value is, the more mismatched the hypothetical model is with the data. Equations 1–3 provide the formulas for evaluating the goodness-of-fit of SEM.


(1) $$\mathrm{TLI}=\frac{\left(T_{b}/{\mathrm{df}}_{b})-(T_{h}/{\mathrm{df}}_{h}\right)}{(T_{b}/{\mathrm{df}}_{b})-1}$$

(2) $$\mathrm{CFI}=\frac{\left(T_{b}-{\mathrm{df}}_{b})-(T_{h}-{\mathrm{df}}_{h}\right)}{\left(T_{b}-{\mathrm{df}}_{b}\right)}$$

(3) $$\text{RMSEA}=\sqrt{\frac{T_{h}-{\mathrm{df}}_{h}}{{\mathrm{df}}_{h}(N-1)}}$$

### 2.3 Statistical analysis

Explanatory variables were selected in this study based on a data-rich environment, and all questions in the questionnaire that all participants could answer were selected (P0 = 141, variables that can only be answered by a specific group were not considered, such as questions only for women: whether they have been screened for cervical cancer). The variable screening and modeling steps can be summarized as follows:

1. Based on the variable-rich environment, the percentage of OMR frequent users and non-users in each variable was summarized. The correlation between each potential explanatory variable and the target variable was explored using the chi-square test of categorical variables and the two-tailed *t*-test of continuous variables. According to the test results, explanatory variables significantly related to the target variable were selected, and the first round of explanatory variable screening was realized (P1 = 44, some variables were merged and answers were regrouped, refer to Supplementary Table 1 for details).
2. The variables selected in the previous round were introduced into the model after balancing the samples by using the random oversampling method, and the importance of variables was calculated using two machine learning methods, RF and GBM. In the order of variable importance, the top 15 important variables calculated using the two methods are listed. To ensure the robustness of the results, the intersection of the important variable sets chosen by the two methods was finally selected as the final explanatory variable set (P2 = 8).
3. Using the structural equation model, the final explanatory variables were categorized into three potential influencing factors (*f* = 3). Then, the relationship and inference path between each influencing factor and the target variable were explored. Based on the results, suggestions for improving the OMR utilization rate were provided.

The structural equation model was modeled using MPlus software (19). All other statistical analyses were performed on R Software (20) version 4.1.2. *p* < 0.05 was considered statistically significant.

## 3 Results

**Figure 2:**
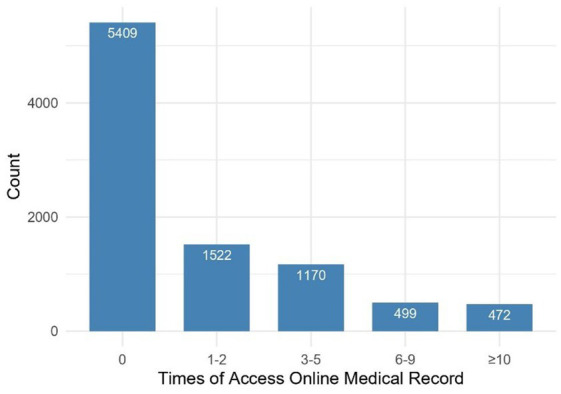
Access to online medical records count, 2019–2020 survey participants.

Figure 2 presents the frequency distribution of the number of visits to OMR in the last 12 months. After removing missing values for the variable “Time of Access to OMR,” the combined dataset from HINTS Cycle 3–Cycle 4 had a total of 9,072 respondents. The study included 971 individuals used OMR frequently and 5,409 individuals who never used OMR. The number of OMR users is relatively high, but most are infrequent users, with a low proportion of frequent users. Specifically, a significant percentage of users have accessed OMR 0–5 times in the past 12 months, while those who have used it more than 5 times represent a smaller share. It is important to note that the respondents in this study do not include those who indicated “1–2 times” and “3–5 times” in Figure 2. This approach allows us to clearly distinguish between frequent users of OMR and those who do not use it, which facilitates our analysis.

This study explored the differences in characteristics between frequent users and non-users of OMR. In this study, the target variable included 971 (15.22%) OMR frequent users and 5,409 (84.78%) OMR non-users. Table 1 lists the frequencies and proportions of the variables. The chi-square test of categorical variables and the *t*-test of continuous variables revealed significant differences in some variables between the frequent users and nonusers (*p* < 0.05). Among sociodemographic variables, the proportion of respondents who chose “female,” “non-Hispanic white,” “>high school,” and “with spouse” was higher in the frequent users group. For example, in the frequent users group, “with spouse” accounted for 64.88%, whereas in the non-users group, the number reduced to 47.73%. In the analysis of second part, the focus was only on frequent users (*n* = 971) and factors regarding their preference for using OMR were explored. The population with a higher proportion of OMR frequent users has higher trust in getting advice or information about health or medical topics, using the internet to find information, paying more attention to his or her health care, and having his or her medical records maintained in a computerized system. For example, in the frequent user group, respondents who had looked for information about cancer accounted for 70.45%, whereas in the non-users group, the percentage decreased to 44.50%. The same was true for the following preferences: “trust information about health or medical topics from government health agencies” (76.98% vs. 68.33%), “use internet” (97.21% vs. 71.41%), “often see a particular doctor, nurse, or other health professional” (88.23% vs. 62.06%), and “have been offered online access to medical records by a health care provider” (96.69% vs. 34.37%). The *t*-test of continuous variables also showed that on some variables such as “age,” “BMI,” “average time sitting,” and “weekly minutes of moderate exercise,” a significant difference was observed between the frequent users and non-users (*p* < 0.05).

To further screen variables and explore the factors influencing frequent OMR use, we introduced the 44 influencing variables and target variables listed in Table 1 into the RF and GLM models. The 44 influencing variables were ranked according to their importance. Table 2 presents the top 15 important variables and importance scores in the two machine learning methods. The main reasons for selecting 15 variables are twofold: first, the remaining variables have significantly lower importance compared to these 15; second, the R software can screen a maximum of 15 variables. All variables selected by the two machine learning methods were classified into seven types of factors (indicated in Table 2): (1) “Whether to provide OMR,” (2) “Degree of concern for health,” (3) “Whether to use internet,” (4) “Psychological conditions,” (5) “Whether to play regular sports,” (6) “Sociodemographic characteristics,” and (7) “Living habits and experience.” In Table 2, we use Roman numerals to represent these seven categories. The Roman numerals in parentheses indicate the category to which each variable belongs. For instance, “Access to OMR_provider (I)” in Table 2 signifies that “Access to OMR_provider” falls under the first category, “Whether to provide OMR.” For the robustness of the results, we chose the intersection of the top 15 importance scores selected by the two methods, and the intersection contained eight variables. These eight variables were categorized into the first three factors among the aforementioned seven factors: (1) “Whether to provide OMR,” (2) “Degree of concern for health,” and (3) “Whether to use internet.”

**Table 2:** Predictors of online medical records nonusers built using random forests and GLM.

Random forests	Importance	GLM	Importance
Predictor		Predictor	
Access to OMR_provider (I)	100.00	Electronic_use (III)	100.00
Freq go provider (II)	51.74	Access to OMR_provider (I)	82.94
Access to OMR_insurer (I)	35.82	Maintained OMR (I)	56.06
Electronic_use (III)	25.12	Freq go provider (II)	46.56
Maintained OMR (I)	22.31	Access to OMR_insurer (I)	20.40
Regular provider (II)	20.22	Shared health info_device (III)	19.13
Shared health info_device (III)	20.03	Regular provider (II)	18.57
Age (VI)	16.12	Weekly minutes moderate exercise (V)	13.04
Average time sitting (V)	15.19	Strength training (V)	11.39
Have device (III)	14.36	Ever had cancer (VII)	9.89
BMI (VI)	14.07	Internet_use (III)	8.91
Influence cancer_obesity (II)	10.67	Race (VI)	8.66
Everything cause cancer (IV)	10.17	Confident_get health info (II)	8.31
Internet_use (III)	9.84	Heard hpv (II)	7.95
Prevent cancer_not possible (IV)	9.43	Social media use (III)	7.59

The fit indices and the significance of the *p*-value of factor loadings revealed that the selected indicators were valid measures of the model factors (Table 3). The factor loadings between OMR availability and the observation variables, namely Maintained OMR (factor loading = 0.927, *p* < 0.01), Access to OMR_provider (factor loading = 1.000, *p* < 0.01), and Access to OMR_insurer (factor loading = 0.536, *p* < 0.01) were significant. This indicates the presence of a sufficient variance explained rate to show that each variable can be represented on the same factor. Similarly, the factor loadings between health care and the observation variables, namely Freq go provider (factor loading = 1.000, *p* < 0.01) and Regular provider (factor loading = 1.110, *p* < 0.01), were significant. The factor loadings between the access to internet to find information and the observation variables, namely Internet_use (factor loading = 0.905, *p* < 0.01), Electronic_use (factor loading = 1.000, *p* < 0.01), and Shared health info_device (factor loading = 0.612, *p* < 0.01), were significant. The factor loading signs were all positive, indicating a positive correlation between the measured and latent variables.

**Table 3:** Factors with their factor loadings on measures from HINTS Cycle 3–Cycle 4.

Factors	Measures	Questions in HINTS Cycle 3–Cycle 4	Factor loading
Whether to provide OMR	Maintained OMR	Do any of your doctors or other health care providers maintain your medical records in a computerized system?	0.927***
	Access to OMR_provider	Have you ever been offered online access to your medical records by your health care provider?	1.000***
	Access to OMR_insurer	Have you ever been offered online access to your medical records by your health insurer?	0.536***
Degree of concern for health	Freq go provider	In the past 12 months, not counting times you went to an emergency room, how many times did you go to a doctor, nurse, or other health professional to get care for yourself?	1.000***
	Regular provider	Not including psychiatrists and other mental health professionals, is there a particular doctor, nurse, or other health professional that you see most often?	1.110***
Whether to use internet	Internet_use	Do you ever go on-line to access the Internet or World Wide Web, or to send and receive e-mail?	0.905***
	Electronic_use	In the past 12 months, have you used a computer, smartphone, or other electronic means to do things on medical activities?	1.000***
	Shared health info_device	Have you shared health information from either an electronic monitoring device or smartphone with a health professional within the last 12 months?	0.612***

***p < 0.001.

Based on the selected eight variables and four assumptions, three measurement models and one structural model were constructed. Moreover, standardized estimates from the WLSMV method from SEM were used to test specific model assumptions.

*Hypothesis 1*: OMR availability should be linked to individuals who tend to use OMR frequently. As shown in Figure 2, OMR availability is measured using three variables: Maintained OMR, Access to OMR_provider, and Access to OMR_insurer. OMR availability is positively related with OMR use (SPC = 0.725, *p* < 0.01).

*Hypothesis 2*: Frequent users of OMR are more likely to be characterized by more concern for health information. The concern for health is measured using two variables: Freq go provider and Regular provider. The higher probability of being frequent users of OMR should be linked to more concern for health (SPC = 0.244, *p* < 0.01).

*Hypothesis 3*: Electronic product usage affects OMR use. Access to internet to find information is measured using three variables: Internet_use, Electronic_use, and Shared health info_device. The higher probability of being frequent users of OMR should be highly linked to access to internet to find information (SPC = 0.413, *p* < 0.01).

*Hypothesis 4*: OMR availability is a mediator variable that the other two factors affect the dependent variable. The two factors “concern for health” and “access to internet to find information” indirectly affect the dependent variable by the mediator variable: OMR availability (Indirect effects = 0.465, *p* < 0.01; Indirect effects = 0.244, *p* < 0.01, respectively).

**Figure 3:**
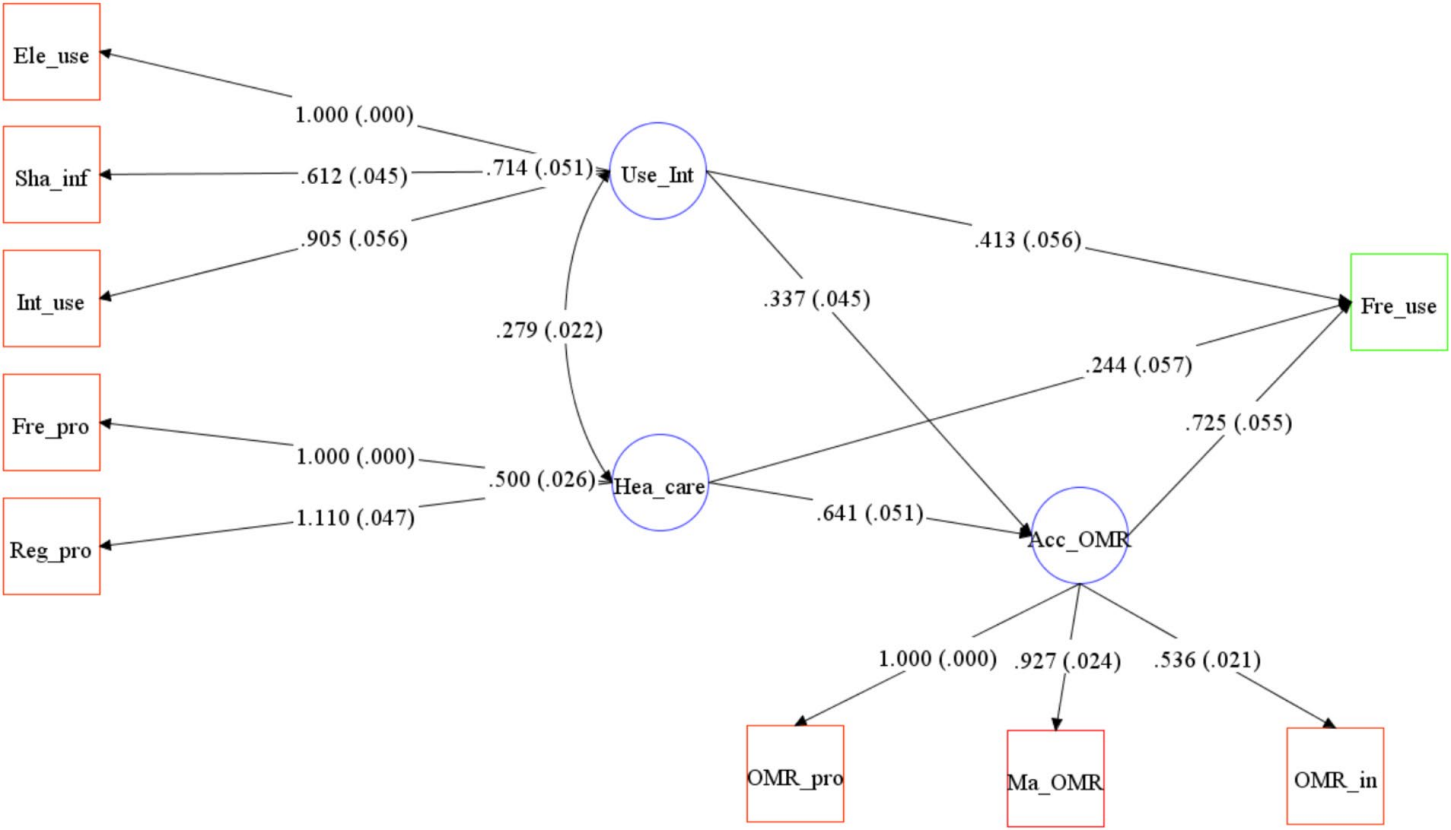
A psychological and behavioral model of frequent use of online medical records.*χ*^2(22)^ = 319.413, *p* < 0.05; Comparative Fit Index (CFI), 0.980; Tucker–Lewis Index (TLI), 0.968; Root Mean Square Error of Approximation (RMSEA), 0.066. Path loadings are standardized coefficients. Note that the indirect effects of “Use_Int” and “Hea_care” on the dependent variable are not depicted in this figure. The impact coefficients for these effects are presented in Hypothesis 4.

The original structural model fits the data well, as shown in Figure 3. The values between factors and dependent variables above the arrows are standardized path coefficients. These coefficients indicate the direct effect of one variable on another. The standard deviations are indicated in parentheses.

## 4 Discussion

The ability of patients or individuals to access their OMR is among the pillars for improving patient engagement and outcomes in the healthcare system. Using nationally representative data from HINTS Cycle 3–Cycle 4, we examined factors influencing OMR usage. Previous survey findings showed that OMR use was improving compared with that in previous years—41% overall in 2019 compared with 28% in 2017 (12). However, the proportion of individuals who regularly use OMR remains low—11% in the combined data for 2019–2020. We attempted to understand why patients tend to using OMR and compared the characteristics of frequent OMR users and OMR non-users based on socio-demographic and questionnaire results.

Among the 6,380 respondents, 971 (15.22%) were frequent users. From a sociodemographic perspective, consistent with past survey findings, respondents who chose “female,” “non-Hispanic white,” “>high school,” and “with spouse” were even more inclined to access OMR. Frequent users are younger than non-users of OMR. Studies have also reported that those older than 65 years would be less likely to use the internet to find health information (21) and less likely to use electronic personal health records (22).

According to the results of the structural equation model, OMR availability is the most direct and crucial factor affecting OMR use. It is also the mediator of the other two influencing factors. In the UK, patients’ access to their own medical records is a critical element of patient centered healthcare. Initiatives to enable patients to access and understand their electronic health records are gaining momentum in the UK, with the 2015 constitution of the National Health Service in England guaranteeing patients access to their health records (23). At present, the rapidly developing electronic information technology can provide the necessary technology for OMR.

Similar to previous findings, people with more health concerns often use OMR. Conversely, lack of attention to one’s health and lack of the need for health-related information can also lead to not using OMR. An ONC (The Office of the National Coordinator for Health Information Technology) study found that individuals may not appreciate the value of accessing their OMR until they have a medical need. Given that the patient record request process can be time consuming, accessing one’s data prior to an urgent health need may be more beneficial (1). Therefore, popularizing health knowledge among the public and increasing public’s attention to health information can to some extent increase the public’s demand for health-related information, thereby promoting OMR use. This paper confirms the positive impact of patient health concerns on the use of OMR, providing health departments with new insights for chronic disease management.

The use of electronic devices is also a factor that influences OMR use. Moreover, people who are proficient in using electronic products find it more convenient to use OMR, and the convenience and readability that OMR offers to these people can be more fully reflected. The reason for avoiding OMR may not be a disadvantage of OMR itself, but rather resistance mentality to electronics. This paper identifies a positive correlation between technology acceptance and the frequency of OMR usage, highlighting the current disparities in digital health access.

Based on the research results, we puts forward several corresponding suggestions. First, healthcare authorities worldwide should recognize and actively promote the adoption of Online Medical Records (OMR). This study underscores the critical role of hospital electronic medical record systems and provides empirical support for governments to prioritize digital infrastructure in primary healthcare institutions. Given resource constraints, the findings suggest that expanding system coverage—particularly by upgrading information systems in underserved and remote hospitals—should take precedence. Once this foundational step is achieved, patient-side engagement initiatives can be introduced to encourage usage. This phased approach offers actionable insights for public health agencies in optimizing financial allocations. Second, integrating electronic medical record systems with health education initiatives could enhance their public health impact. For instance, the system could automatically deliver personalized health recommendations (e.g., for hypertension management) to patients upon accessing their records. By leveraging data analytics to proactively identify high-need populations, healthcare providers can deliver more precise and effective interventions, thereby improving health outcomes at scale. Third, policy measures should be supplemented with community-based digital literacy programs and alternative access solutions to ensure equitable adoption. Not all patients may have access to or proficiency with smartphones; thus, supplementary channels—such as telephone-based inquiry systems—should be implemented to bridge the digital divide and maximize inclusivity in healthcare delivery.

Based on the data from the National Cancer Institute’s 2019–2020 HINTS database, this study identified factors influencing patients’ predisposition to use OMR. Moreover, this study compared the characteristics of frequent users and non-users of OMR based on demographic and other factors. Using a combination of traditional statistical tests and two machine learning algorithms, we identified 8 variables as key variables in the frequent use of OMR and divided them into three influencing factors (latent variables). The structural equation model was used to conduct the inference path of the three influencing factors and target variables. The three impact factors were (1) “Whether to provide OMR,” (2) “Degree of concern for health,” (3) “Whether to use internet.” This paper proposes recommendations based on the three impact factors, thereby promoting the usefulness of medical records in a larger group of people. This article also has some limitations. For instance, it does not conduct an in-depth analysis of whether the influencing factors of online medical record (OMR) usage frequency are consistent across different populations. Additionally, some of the factors analyzed rely on patient self-assessment, which may introduce social expectation bias or cognitive errors. In future studies, we will expand the diversity of our sample by including patients from different regions, socioeconomic backgrounds, and age groups, with particular attention to the usage barriers faced by digitally disadvantaged populations. Furthermore, we will investigate whether the mechanisms influencing OMR adoption have shifted in the post-pandemic era compared to pre-pandemic trends. Additionally, this research will conduct cross-system comparisons to examine how drivers of electronic medical record utilization vary across different healthcare systems, aiming to derive universally applicable principles for OMR implementation and optimization.

## Data Availability

Publicly available datasets were analyzed in this study. This data can be found at: HINTS database, https://hints.cancer.gov/.

## References

[1] Patel V. Johnson C. Individuals’ use of online medical records and technology for health needs. ONC Data Brief No. 40 (2018), pp. 1–12.

[2] Guo K Austin M De Mendonca B Cantor Z Wall M Cox C. Evaluating the impact of a specialized and centralized online medical consultation system for paramedics: pilot study. Can J Emerg Med. (2025) 27:38–42. doi: 10.1007/s43678-024-00792-3, PMID: 39382769

[3] Derecho KC Cafino R Aquino-Cafino SL Isla A Jr Esencia JA Lactuan NJ. Technology adoption of electronic medical records in developing economies: a systematic review on physicians’ perspective. Digit Health. (2024) 10:20552076231224605. doi: 10.1177/20552076231224605, PMID: 38222081 PMC10787531

[4] Fleming SL Lozano A Haberkorn WJ Jindal JA Reis E Thapa R. Medalign: a clinician-generated dataset for instruction following with electronic medical records. Proc AAAI Conf Artif Intel. (2024) 38:22021–30. doi: 10.1609/aaai.v38i20.30205 PMC12826664 41584261

[5] Norouzinia R Aghabarari M Shiri M Karimi M Samami E. Communication barriers perceived by nurses and patients. Glob J Health Sci. (2016) 8:65. doi: 10.5539/gjhs.v8n6p65 PMC4954910 26755475

[6] Lloyd S Long K Probst Y Di Donato J Oshni Alvandi A Roach J. Medical and nursing clinician perspectives on the usability of the hospital electronic medical record: a qualitative analysis. Health Inf Manag J. (2024) 53:189–97. doi: 10.1177/18333583231154624, PMID: 36866778 PMC11401339

[7] Hogan TP Wakefield B Nazi KM Houston TK Weaver FM. Promoting access through complementary eHealth technologies: recommendations for VA’S home telehealth and personal health record programs. J Gen Intern Med. (2011) 26:628–35. doi: 10.1007/s11606-011-1765-y, PMID: 21989614 PMC3191221

[8] Umefjord G Hamberg K Malker H Petersson G. The use of an internet-based ask the doctor service involving family physicians: evaluation by a web survey. Fam Pract. (2006) 23:159–66. doi: 10.1093/fampra/cmi117, PMID: 16464871

[9] Umefjord G Malker H Olofsson N Hensjö LO Petersson G. Primary care phycians’ experiences of carrying out consultations on the internet. Inform Prim Care. (2004) 12:85–90. doi: 10.14236/jhi.v12i2.112 15319060

[10] Chou WYS Hunt YM Beckjord EB Moser RP Hesse BW. Social media use in the United States: implications for health communication. J Med Internet Res. (2009) 11:e1249. doi: 10.2196/jmir.1249 PMC2802563 19945947

[11] Shah SGS Fitton R Hannan A Fisher B Young T Barnett J. Accessing personal medical records online: a means to what ends? Int J Med Inform. (2015) 84:111–8. doi: 10.1016/j.ijmedinf.2014.10.005, PMID: 25453275

[12] Elkefi S Yu Z Asan O. Online medical record nonuse among patients: data analysis study of the 2019 health information national trends survey. J Med Internet Res. (2021) 23:e24767. doi: 10.2196/24767, PMID: 33616539 PMC7939938

[13] Nelson D Kreps G Hesse B Croyle R Willis G Arora N. The health information national trends survey (HINTS): development, design, and dissemination. J Health Commun. (2004) 9:443–60. doi: 10.1080/10810730490504233 15513791

[14] Breiman L. Random forests. Mach Learn. (2001) 45:5–32. doi: 10.1023/A:1010933404324

[15] Nykodym T Kraljevic T Wang A Wong W. Generalized linear modeling with H2O. California, USA: H2O ai, Inc. (2016).

[16] Anderson JC Gerbing DW. Structural equation modeling in practice: a review and recommended two-step approach. Psychol Bull. (1988) 103:411–23. doi: 10.1037/0033-2909.103.3.411

[17] Hoyle RH. Structural equation modeling: concepts, issues, and applications. London, UK: Sage (1995).

[18] Browne MW Cudeck R. Alternative ways of assessing model fit. Sociol Methods Res. (1992) 21:230–58. doi: 10.1177/0049124192021002005

[19] Muthén LK Muthén BO. Mplus user’s guide. Sixth ed. Los Angeles: (2007).

[20] R Core Team. (2021). R: a language and environment for statistical computing. R Foundation for Statistical Computing, Vienna, Austria. Available online at: https://www.R-project.org/ (Accessed July 24, 2025).

[21] Asan O Cooper F II Nagavally S Walker RJ Williams JS Ozieh MN. Preferences for health information technologies among US adults: analysis of the health information National Trends Survey. J Med Internet Res. (2018) 20:e9436. doi: 10.2196/jmir.9436, PMID: 30341048 PMC6245956

[22] Wen KY Kreps G Zhu F Miller S. Consumers’ perceptions about and use of the internet for personal health records and health information exchange: analysis of the 2007 health information national trends survey. J Med Internet Res. (2010) 12:e1668. doi: 10.2196/jmir.1668 PMC3056530 21169163

[23] Pagliari C Shand T Fisher B. Embedding online patient record access in UK primary care: a survey of stakeholder experiences. JRSM Short Rep. (2012) 3:1–7. doi: 10.1258/shorts.2012.012009, PMID: 22666531 PMC3365791

