# Supplementary material for: Frequent use of online medical records: analysis of influence factors based on structural equation modeling
Source: Front Public Health. 2025 Aug 20;13:1609503. doi: 10.3389/fpubh.2025.1609503 (PMC12405286; doi:10.3389/fpubh.2025.1609503)
Supplement: Supplementary file 1 [file Table_1.DOCX]

Supplementary Material

# Supplementary Tables

Supplementary Material Table 1. Variables extracted from the HINTS database for research

| **Variable** | **Questions from the HINTS** | **Options and coding** | **Coding used in this article** |
| --- | --- | --- | --- |
| **Dependent variable** | | | |
| use of online medical records | How many times did you access your online medical record in the last 12 months? | 0. 0  1. 1 to 2 times  2. 3 to 5 times  3. 6 to 9 times  4. 10 or more times | Frequent user:  3, 4  Non-user:  0 |
| **Demographic variables** | | | |
| Gender | Selfgender | Male  Female | Male  Female |
| Race | What is your race? One or more categories may be selected. | White;  Mexican;  PuertoRican;  Cuban;  NotHisp;  OthHisp;  AmerInd Black;  AsInd;  Chinese;  Filipino;  OthAsian;  OthPacIsl;  1. Yes  2. No | Non-Hispanic white:  White=1&others=2  Racial and ethnic minority:  others |
| Education | Education: What is the highest grade or level of schooling you completed? | 1. Less than 8 years  2. 8 through 11 years  3. 12 years or completed high school  4. Post high school training other than college  (vocational or technical)  5. Some college  6. College graduate  7. Postgraduate | >High school:  4, 5, 6, 7  ≤High school:  1, 2, 3 |
| Income | IncomeRanges: Thinking about members of your family living in this household, what is your combined annual income, meaning the total pre-tax income from all sources earned in the past year? | 1. $0 to $9,999  2. $10,000 to $14,999  3. $15,000 to $19,999  4. $20,000 to $34,999  5. $35,000 to $49,999  6. $50,000 to $74,999  7. $75,000 to $99,999  8. $100,000 to $199,999  9. $200,000 or more | ≥$20,000:  4, 5, 6, 7, 8, 9  <$20,000:  1, 2, 3 |
| Incomefeelings | Incomefeelings: Which one of these comes closest to your own feelings about your household income? | 1.Living comfortably on present income  2.Getting by on present income  3.Finding it difficult on present income  4.Finding it very difficult on present income | Not difficult:  1, 2  Difficult:  3, 4 |
| Area | RUC2013: USDA Rural/Urban Designation (2013) | codes 1–9 | Metropolitan:  1–3  Non-metropolitan:  4–9 |
| Marital status | MaritalStatus: What is your marital status? | 1. Married  2. Living as married or living with a romantic  partner  3. Divorced  4. Widowed  5. Separated  6.Single, never been married | In marriage:  1, 2  Not in marriage:  3, 4, 5, 6 |
| Age | SelfAge | SelfAge___ | **____** |
| BMI | Body Mass Index | (Weight*703)/(Height in inches**2) | (Weight*703)/(Height in inches**2) |
| **Other related variables** | | | |
| Confident_get health info | ConfidentGetHealthInf: Overall, how confident are you that you could get advice or information about health or medical topics if you needed it? | 1. Completely confident  2. Very confident  3. Somewhat confident  4. A little confident  5. Not confident at all | High_level:  1, 2, 3  Low_level:  4, 5 |
| Trust_doctor | TrustDoctor: In general, how much would you trust information about health or medical topics from a doctor? | 1. A lot 2. Some 3. A little 4. Not at all | High_level:  1, 2  Low_level:  3, 4 |
| Trust_gov | TrustGov: In general, how much would you trust information about health or medical topics from government health agencies? | 1. A lot 2. Some 3. A little 4. Not at all | High_level:  1, 2  Low_level:  3, 4 |
| Trust_charities | TrustCharities: In general, how much would you trust information about health or medical topics from charitable organizations? | 1. A lot 2. Some 3. A little 4. Not at all | High_level:  1, 2  Low_level:  3, 4 |
| Trust_religiousOrgs | TrustReligiousOrgs: In general, how much would you trust information about health or medical topics from religious organizations and leaders? | 1. A lot 2. Some 3. A little 4. Not at all | High_level:  1, 2  Low_level:  3, 4 |
| Electronic_use | Electronic_use: In the past 12 months, have you used a computer, smartphone, or other electronic means to do any of the following?  a. Looked for health or medical information for yourself  b. Bought medicine or vitamins online  c. Used e-mail or the Internet to communicate with a doctor or a doctor’s office  d. Tracked health care charges and costs  e. Looked up medical test results  f. Made appointments with a health care provider  g. Looked for information about the harms of electronic or e-cigarettes (also known as vapes, vape-pens, tanks, mods, or pod-mods) | 1. Yes  2. No | Yes:  a=1 or b=1 or c=1 or d=1 or e=1 or f=1 or g=1  No:  a=2& b=2& c=2& d=2& e=2& f=2& g=2 |
| Social media use | Intrsn_use: In the past 12 months, have you used the Internet for any of the following reasons?  a. To visit a social networking site, such as Facebook or LinkedIn  b. To share health information on social networking sites, such as Facebook or Twitter  c. To write in an online diary or blog (i.e., Web log)  d. To participate in an online forum or support group for people with a similar health or medical issue  e. To watch a health-related video on YouTube | 1. Yes  2. No | Yes:  a=1 or b=1 or c=1 or d=1 or e=1  No:  a=2& b=2& c=2& d=2& e=2 |
| Insurance | Healthins_use: Are you currently covered by any of the following types of health insurance or health coverage plans?  a. Insurance through a current or former employer or union  b. Insurance purchased directly from an insurance company  c. Medicare, for people 65 and older, or people with certain disabilities  d. Medicaid, Medical Assistance, or any kind of government-assistance plan for those with low incomes or a disability  e. TRICARE or other military health care  f. VA (including those who have ever used  or enrolled for VA health care)  g. Indian Health Service  h. Any other type of health insurance or health coverage plan (Specify) | 1. Yes  2. No | Yes:  a=1 or b=1 or c=1 or d=1 or e=1 or f=1 or g=1 or h=1  No:  a=2& b=2& c=2& d=2& e=2& f=2& g=2& h=2 |
| Caregiving who | CaregivingWho_Cat: Are you currently caring for or making health care decisions for someone with a medical, behavioral, disability, or other condition?  a. Yes, a child/children  b. Yes, a spouse/partner  c. Yes, a parent/parents  d. Yes, another family member  e. Yes, a friend or other non-relative  f. No | 1. Selected 2. Not Selected | Yes:  a=1 or b=1 or c=1 or d=1 or e=1  No:  f=1 |
| Generalhealth | GeneralHealth: In general, would you say your health is... | 1. Excellent,  2. Very good,  3. Good,  4. Fair, or  5. Poor? | Relative Good:  1, 2, 3  Relative Bad:  4, 5 |
| Ownabilitytakecarehealth | OwnAbilityTakeCareHealth: Overall, how confident are you about your ability to take good care of your health? | 1. Completely confident  2. Very confident  3. Somewhat confident  4. A little confident  5. Not confident at all | High_level:  1, 2, 3  Low_level:  4, 5 |
| Drinkdays per week | DrinkDaysPerWeek: During the past 30 days, how many days per week did you have at least one drink of any alcoholic beverage? | ___Days per week | None:  0  1-3  4-7 |
| Moderate exercise | TimesModerateExercise: In a typical week, how many days do you do any physical activity or exercise of at least moderate intensity? | ___Days per week | None:  0  1-3 days per week  4-7 days per week |
| Strength training | TimesStrengthTraining: In a typical week, outside of your job or work around the house, how many days do you do leisure-time physical activities specifically designed to strengthen your muscles? | ___Days per week | None:  0  1-3 days per week  4-7 days per week |
| Electronic cigarettes | Electciglessharm: Compared to smoking cigarettes, would you say that electronic cigarettes are... | 1. Much less harmful 2. Less harmful 3. Just as harmful 4. More harmful 5. Much more harmful, or 6. I don't know | relative less harmful:  1, 2, 3  relative more harmful:  4, 5  I don't know:  6 |
